# Characterization of the Phenolic Profile and Antioxidant Activity of *Cathissa reverchonii* (Lange) Speta

**DOI:** 10.3390/molecules27061979

**Published:** 2022-03-18

**Authors:** Eulogio J. Llorent-Martínez, Ana I. Gordo-Moreno, María Luisa Fernández-de Córdova, Carlos Salazar-Mendías, Amanda Tercero-Araque

**Affiliations:** 1 Department of Physical and Analytical Chemistry, Faculty of Experimental Sciences, University of Jaén, Campus Las Lagunillas, E-23071 Jaén, Spain; aigm0010@red.ujaen.es (A.I.G.-M.); mferna@ujaen.es (M.L.F.-d.C.); 2 Department of Animal Biology, Plant Biology and Ecology, Faculty of Experimental Sciences, University of Jaén, Campus Las Lagunillas, E-23071 Jaén, Spain; csalazar@ujaen.es (C.S.-M.); ata00016@red.ujaen.es (A.T.-A.)

**Keywords:** phenolic, saponin, *Cathissa*, environmental factors, HPLC-MS

## Abstract

*Cathissa reverchonii* (formerly *Ornithogalum reverchonii*) is a threatened species, constituting an endemism present in the south of Spain and northern Morocco. In Spain, it is only found in two disjoint populations in the region of Andalusia. The determination of its chemical composition and the influence that environmental factors have on it can contribute significantly to the development of appropriate protection and conservation plans. However, there are no previous reports about this species to date. Consequently, this research aimed to study the phenolic composition and antioxidant activity of *C. reverchonii* and to assess the influence of environmental factors on the phenolic profile and bioactivity. The vegetal material was collected in seven places inhabited by the two separate populations in Spain. The phenolic composition of methanolic extracts of the species was determined by HPLC-ESI-Q-TOF-MS, and the antioxidant activity was assessed by DPPH and ABTS assays. Fifteen compounds were characterized in the extracts of the aerial parts of *C. reverchonii*, revealing differences in the phytochemical profile between both populations analyzed, mainly in the saponin fraction. The main phenolics were flavone di-*C*-glucoside (lucenin-2), followed by a quercetin-di-*C*-glucoside. The composition of the extracts of *C. reverchonii* and their radical scavenging power were compared with those of other species of the genus *Ornithogalum* L., revealing significant differences between the latter and the genus *Cathissa*.

## 1. Introduction

Plants and plant-derived products are important sources of many natural compounds, such as phenolics, with health-promoting activities. Hence, exhaustive research has been conducted in the last decade to report the phytochemical profiles and bioactivity of understudied, unknown, or threatened plant species as potential sources of bioactive compounds for the pharmaceutical and food industries. In this manuscript, we have focused our investigation on the plant *Cathissa reverchonii* (Lange) Speta (=*Ornithogalum reverchonii* Lange).

The genus *Cathissa* (Salisb.) Baker is a monophyletic group consisting of only three species distributed in southwestern areas of the Iberian Peninsula (Spain) and northern Morocco: *C. broteroi* (M. Laínz) Speta, *C. concinna* (Salisb.) Speta and *C. reverchonii* (Lange) Speta. This genus is taxonomically included in the order Asparagales Link, subfamily Scilloideae Burnett, and family Asparagaceae Jussieu [1].

**Figure 1:**
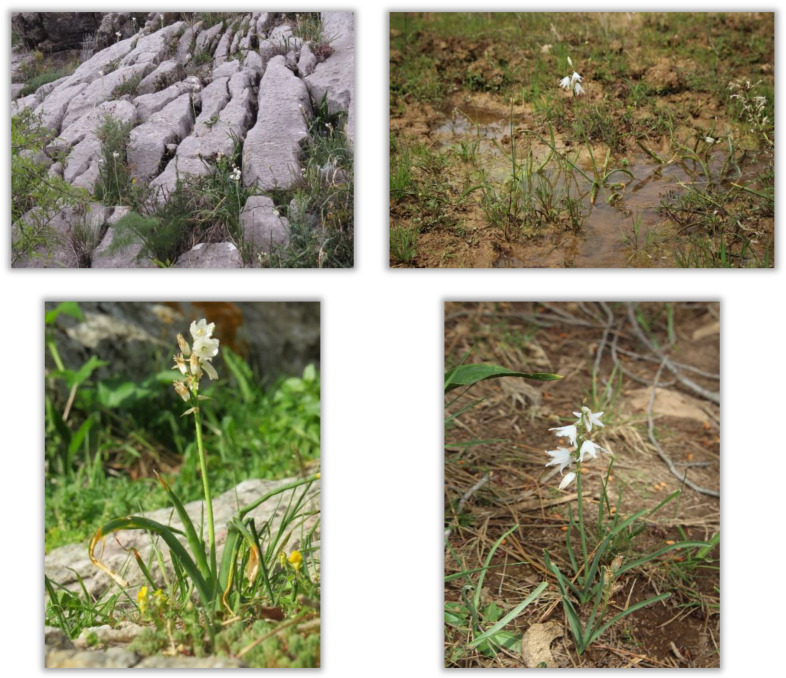
Photographs of the plant species (author: A.T.-A.). Western population: ALP (**upper left**) and VIL (**bottom left**). Eastern population: LV2 (**upper right**) and LV3 (**bottom right**). See Table 1 for acronyms and information on each location.

*Cathissa* has been treated both as a section [2] and as a subgenus [3] of the genus *Ornithogalum* L. After conducting phylogenetic studies, based on plastidial and nuclear sequences, it was considered as an independent genus [4]. *C. reverchonii* is a Baetic-Riffean endemic species present in southern Spain and northern Morocco. Specifically, in Spain, there are two populations: a western one (provinces of Cádiz and Málaga) and an eastern disjunction (province of Jaén) [5]. It develops on calcareous rocks [6] sheltering in shady and humid crevices [7]. However, in the eastern population, it grows on relatively humid clay soils, among limestone rocks, with little or no inclination, as seems to be the case in the localities of Morocco. Photographs of the plant species of the two populations are shown in Figure 1.

*C. reverchonii* is a threatened and legally protected species in Spain (and Europe), granting it status in the “NT” IUCN category (“near threatened”) [5]. In the legal documents, it is found at the international level as “Vulnerable” in the European Habitats Directive (92/43/EEC) [8], at the Spanish level as “Special Protection Regime” [9], and at the Andalusian regional level as “Vulnerable” in the Andalusian Endangered Species Catalogue [10]. According to the European Habitats Directive, the Member States must undertake conservation measures to maintain plant species and their habitats in a “favourable conservation status”, implying, if necessary, appropriate management plans. The high level of plant diversity of threatened species represents a considerable challenge for conservation and, therefore, the elaboration of management plans. Plant species often exist only in a small area, and populations are normally isolated; surveillance or scientific data are also frequently lacking. Therefore, knowledge of the species present in a certain territory is essential to develop coherent conservation policies. 

There are previous studies regarding the chemical profile and antioxidant activity of *Ornithogalum*, to which *C. reverchonii* was considered to belong until 2001 [11]. For instance, the phenolic profile composition and bioactivity of *O. sigmoideum*, *O. orthophyllum*, *O. oligophyllum*, *O. lancelatum,* and *O. narbonense* have been reported [12,13,14]. However, this work is the first report in the literature studying the phytochemical composition and biological activity of the genus *Cathissa*. Hence, the aims of the present study were a) to evaluate the chemical composition of *C. reverchonii* by HPLC-ESI-Q-TOF-MS; (b) to study its antioxidant activity; and (c) to evaluate whether environmental and phytogeographical differences influence the chemical composition and antioxidant activity of the two different populations of this species in Spain.

## 2. Results and Discussion

### 2.1. Characterization of Phytochemicals in Methanolic Extracts

Fifteen compounds were identified or tentatively characterized in the extracts of *C. reverchonii* aerial parts. Accurate mass data, ion source fragmentation, MS/MS fragmentation patterns, as well as METLIN and bibliographic searches were used for characterization. Table 2 contains the compounds identified in negative ionization mode, as well as retention times, experimental [M-H]^-^, molecular formulas, calculated mass error (ppm), and fragment ions.

Compound **2** was identified utilizing an analytical standard, whereas compounds **1** and **3** were identified by comparison of the MS/MS spectra with the METLIN database. 

The molecular formulas of all flavonoids were compared with the METLIN database. In addition, the following information was used to complete the identification. The fragmentation pattern of compound **4** presented fragment ions at *m*/*z* [M−H-60]^−^, [M−H-90]^−^, [M−H-120]^−^, [M−H-210]^−^ and [M−H-210]^−^, consistent with flavonoid di-*C*-glycosides [15]. In the same way, compound **5** was identified as lucenin-2 [16]. Compound **6** was identified as luteolin-6-*C*-glucoside (instead of 8-*C*-glucoside) due to the presence of the fragment ion at m/z 429, [M-H-18]^-^, which is absent in the 8-*C*-glucoside [17]. Similarly, compound **7** was identified as apigenin-8-*C*-glucoside [17].

Compounds **8**, **9**, **11**, **12**, **13**, and **14** were tentatively characterized as saponins due to their molecular formulas, fragmentation patterns, and high retention times. In all cases, neutral losses of 132 (pentoside), 146 (deoxyhexoside), and 162 (hexoside) Da were observed. Compounds **8** and **9** were reported in [18]; compounds **12** and **13** in [19], and compound **14** presented a hexoside moiety less than that of **12** and **13**.

Compounds **15** and **17** were characterized as oxylipins by comparison of the molecular formulas and mass fragmentation information [20].

**Figure 2:**
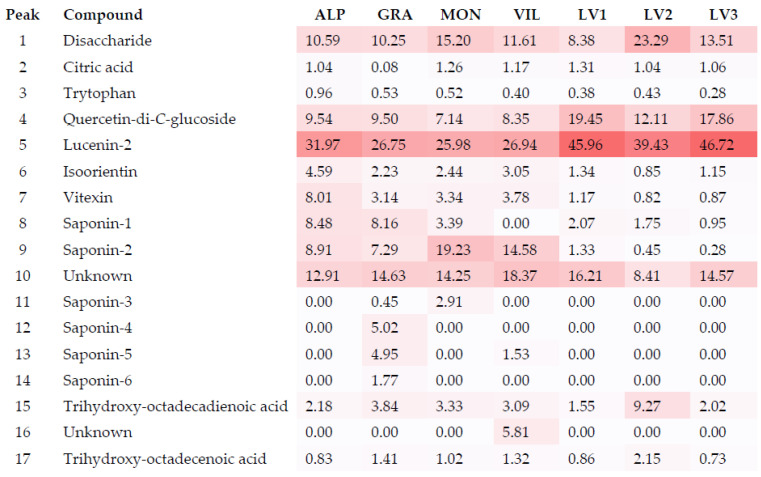
Relative peak areas and heat map obtained by HPLC-Q-TOF-MS analysis of extracts of *C. reverchonii*.

To check which compounds were the most abundant in each extract, peak areas of each compound were obtained by HPLC-Q-TOF-MS in MS mode using the precursor ion [M-H]^-^. The relative percentage of each compound was calculated and is shown in Figure 2, in which the heat map highlights the most abundant compounds (the darker the color, the higher the concentration). 

First of all, there was a major difference regarding the percentage of saponins (compounds **8**, **9**, **11**, **12**, **13,** and **14**) and flavonoids (compounds **4**–**7**) in the two analyzed populations. From Figure 2, it can be observed that the total percentage of saponins was 16–28% in the western population (ALP, GRA, MON, and VIL), compared to less than 4% in the eastern population (LV samples). In addition, although flavonoids were the main compounds in all extracts, the percentages were also different among the two populations: 39–54% and 53–68% in the western and eastern populations, respectively.

Regarding individual compounds, it can be observed that the major component and active principle of *C. reverchonii* was identified as the flavone di-*C*-glucoside, lucenin-2 (compound **5**) in all the samples analyzed. Other *C*-glycosyl flavonoids (compounds **4**, **6** and **7**) were also present in significant amounts in *C. reverchonii* extracts. Flavonoid *C*-glucosides, much less common than *O*-glucosides, are absorbed unchanged in the intestine, distributed to other tissues, and undergo enterohepatic recirculation in addition to hydrolysis, conjugation, and reduction to form a bioavailable glucuronide [21,22]. Flavonoid *C*-glycosides have also been shown to have antibacterial activity [23], antioxidant properties [16], anti-inflammatory activity [24], and anti-hepatotoxic activity [25]. A quercetin-*C*-glucoside (compound **4**) was also identified in the *C. reverchonii* extracts in significant amounts; these glycosides are a type of quercetin derivative that occurs relatively rarely in nature.

Six saponins were also characterized in the extracts, although in low levels compared to flavonoids, except compounds **8** and **9**. Saponins are a vast group of glycosides, widely distributed in higher plants. They show a wide range of biological properties due to their amphiphilic nature. It seems that the natural role of these compounds in plants is to protect against attack by possible pathogens, which would explain their antimicrobial activity [26]. Saponins are also toxic to cool-blooded organisms, and therefore, they can be used as insecticides, antibiotics, and fungicides [27].

Finally, two of the compounds found in *C. reverchonii* extracts were identified as oxylipins (compounds **15** and **17**), a group of biologically active compounds coming from the oxidative metabolism of polyunsaturated fatty acids (PUFA). There is increasing evidence that the collective biological importance of these compounds in plants is comparable to that of the eicosanoid family of lipid mediators in animals [28].

### 2.2. Quantification of Phenolic Compounds

Four flavonoids were quantified in the extracts of *C. reverchonii* by HPLC-DAD (Table 3). Lucenin-2 presented the highest concentration (4.8–8.9 mg g^−1^ DE). This compound represented between 64% and 75% of the total individual phenolic content (TIPC), defined as the sum of all individual phenolic concentrations. 

Since there are no previous reports in the literature about the phenolic profile of *C. reverchonii* or the other two species of the genus *Cathissa*—*C. broteroi* and *C. concinna*—a comparison was made with the phenolic composition of species of the genus *Ornithogalum* L., in which the species *C. reverchonii* was for years classified. From the literature, the most abundant compounds in methanol extracts of aerial parts of the species *O. sigmoideum, O. orthophyllum,* and *O. oligophyllum* were protocatechuic acid, *p*-hydroxybenzoic acid, vanillic acid, and *p*-coumaric acid [13]. In the species *O. armeniacum* and *O. lanceolatum* the major phenolic components found were (+)-catechin and gallic acids [29]. In a latter study [14], it was found that in ethanolic extracts of *O. lanceolatum* protocatechuic acid was the main compound in both plant parts (aerial and bulbs) and that rutin, quercetin, and *p*-coumaric acid were the most predominant phenolic compounds in the aerial parts. Concerning the species *O. narbonense*, the major phenolic compounds were benzoic acid, *p*-hydroxybenzoic acid, chlorogenic acid, rutin, and caffeic acid [12]. These results seem to support that the species *C. reverchonii* does not belong to the genus *Ornithogalum* L., in which it was initially included [11].

For the LV1 and LV3 samples (population 2), a clear correlation was observed between the TIPC values (13.1 ± 0.6 mg g^−1^ DE and 9.8 ± 0.4 mg g^−1^ DE, respectively), which were the highest of all the samples, and the contents of lucenin-2 (8.9 ± 0.5 mg g^−1^ DE and 7.2 ± 0.4 mg g^−1^ DE, respectively) and quercetin-di-C-glucoside (3.8 ± 0.3 mg g^−1^ DE and 2.4 ± 0.2 mg g^−1^ DE, respectively), which were also the highest (Table 3).

### 2.3. Antioxidant Properties

The ability of *C. reverchonii* extracts to act as natural antioxidants was assessed through DPPH^·^ (1,1-diphenyl-2-picrylhydrazyl) and ABTS^·+^ (2,2′-azino-bis(3-ethylbenzothiazoline-6-sulfonic acid) radicals scavenging assays. The results are summarized in Table 4. 

The observed antioxidant capacity of *C. reverchonii* extracts could be explained by the presence of lucenin-2 and quercetin-di-C-glucoside. For the LV1 sample, a clear correlation was observed between the TIPC value (13.1 ± 0.6 mg g^−1^ DE), which was the highest of all the samples, and the ABTS and DPPH values (2.9 ± 0.4 g TE/100 g DE and 2.0 ± 0.3 g TE/100 g DE, respectively), which were also the highest (Table 3 and Table 4). It is difficult to compare the antioxidant activity of *C. reverchonii* (*Cathissa* genus) with other species of the *Ornithogalum* L. genus, as there are few studies in the literature. The radical scavenging power of the species *O. narbonense* and *O. orthophyllum* against ABTS radical was 0.02 ± 0.10 (g TE/100 g DE) [12] and 10.0 ± 1.0 (g TE/100 g DE) [30], respectively. On the other hand, the species *O. narborense* showed an antioxidant activity against DPPH radical of 1.66 ± 0.03 (g TE/100 g DE) [12].

### 2.4. Differences between Both Populations

Regarding the influence of environmental factors on the composition of the extracts, it was found that the samples located in population 2 (LV1, LV2, and LV3) presented a higher content of lucenin-2 and quercetin-di-*C*-glucoside and a lower content of isoorientin and vitexin than those of the population 1 samples (ALP, GRA, MON, and VIL). In addition, as mentioned before, there were big differences regarding the saponin profiles between both populations (saponins were more abundant in population 1). These differences could be attributed to the fact that both populations grow in different conditions of temperature (T), relative humidity (RH), soil composition, precipitation (P), and vegetation (Table 1). Data in Table 1 correspond to 2020, as there are no updated data for the complete year 2021, during which the samples were collected.

Differences were also observed within the same population. When compared with the samples LV1 and LV3, the sample LV2 had the lowest TIPC due to its lower content of lucenin-2 and quercetin-di-*C*-glucoside. Although the same humidity levels are shown in Table 1 for all LV samples, that fact is due to the proximity of locations, so the humidity provided by the website is the same. However, photographs of the locations are shown in Figure 1. It can be observed that in the location of LV2, the water accumulates, which increases the humidity of the LV2 zone. In fact, the plant formations in the areas are dry grassland in LV1 and LV3 and wet grassland in LV2. Hence, climatology is also important even for plants collected in the same population and must be considered to obtain an overall profile of any plant species.

## 3. Materials and Methods

### 3.1. Plant Materials

**Figure 3:**
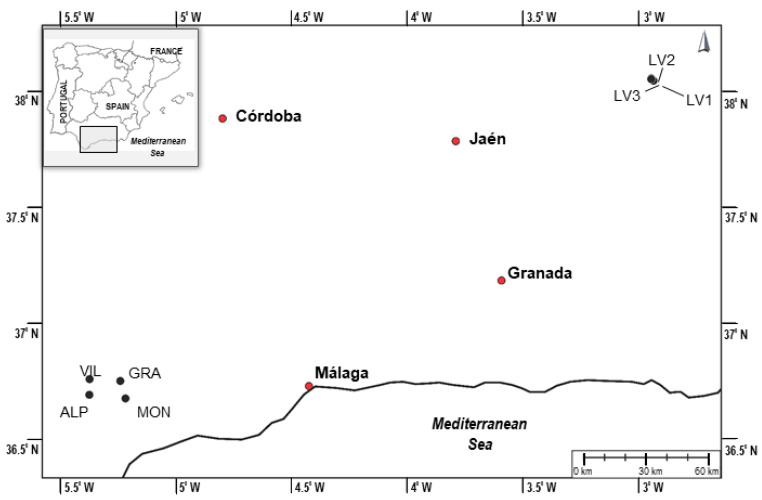
Geographic locations of the sampling sites of *C. reverchonii* species in Andalusia (Spain).

Plants of the species *C. reverchonii* were randomly collected in March and April 2021 in different locations in the south of Spain, where this species has two populations (Table 1 and Figure 3). Botanical authentication was carried out by the botanist Dr. Carlos Salazar Mendías (Department of Animal Biology, Plant Biology, and Ecology of the University of Jaén, Spain). Only the aerial parts of the plants were selected; then, they were washed with ultrapure water and stored in a freezer at −80 °C until use.

### 3.2. Extraction Techniques

Extractions were carried out in methanol (MeOH; HPLC grade). The aerial parts of the plants were lyophilized (ModulyoD/23, Thermo Savant; Waltham, MA, USA) and crushed with a grinder. Extraction was performed as follows: 2.5 g of dry material was extracted with 50 mL MeOH in an ultrasonic liquid processor (Qsonica Sonicators; Newton, CT, USA) with a power of 55 W and a frequency of 20 kHz, for 10 min (using 50% power) at room temperature. Extractions were performed in triplicate. After sonication, solutions were filtered through Whatman No.1 filters. The solvent was evaporated under reduced pressure in a Hei-Vap Precision rotary evaporator (Heidolf; Schwabach; Germany) at 40 °C. Dried extracts (DE) were stored at −20 °C until analysis. 

### 3.3. Antioxidant Capacity

For assessing antioxidant capacity, we used radical quenching power (ABTS and DPPH). The methods’ details were described in our earlier paper [31]. The results were given as standard compound equivalents of Trolox (TE), in g TE/100 g DE. 

### 3.4. Analysis of Phenolic Compounds by HPLC-ESI-Q-TOF-MS

For the analysis of compounds, 5 mg of DE were dissolved in 1 mL MeOH, filtered through 0.45 µm filters, and 10 µL was injected in the HPLC system. Analyses were performed in an Agilent 1200 (Agilent Technologies, Santa Clara, CA, USA) equipped with an Agilent 6530B quadrupole-time-of-flight mass spectrometer (Q-TOF MS). The column used was a Luna Omega Polar C18 of 150 × 3.0 mm and 5 µm particle size (Phenomenex, Torrance, CA, USA). The separation was carried out at room temperature at a flow rate of 0.4 mL min^−1^. The mobile phases were water + formic acid 0.1 % *v*/*v* (eluent A) and acetonitrile (eluent B). The gradient elution was: 10–25% B in 0–25 min, 25% B in 25–30 min, 25–50% B in 30–40 min, 50–100% B in 40–42 min, 100% in 42–47 min. Then, eluent B was returned to 10% with a 7 min stabilization time. To obtain the MS and MS/MS spectra, the mass spectrometer was operated in negative ion mode using an orthogonal ESI source. The MS parameters were: capillary voltage, 3500 V; nebulizer pressure of 45 psi; drying gas flow rate, 10 L/min; gas temperature, 325 °C; skimmer voltage, 60 V; fragmentation voltage, 140 V. The MS and Auto MS/MS modes were set to acquire *m*/*z* values ranging between 50–1200, at a scan rate of 2 and 3 spectra per second, respectively. Agilent Mass Hunter Qualitative analysis software version B.06.00 was used for post-acquisition data processing. 

### 3.5. Quantification of Main Compounds by HPLC-DAD

For the quantitation of the main compounds using UV detection, the HPLC system was an Agilent Series 1100 with a G1315B diode array detector and an ion trap mass spectrometer (Esquire 6000, Bruker Daltonics) equipped with an electrospray interface. The same chromatographic conditions mentioned above were used. Standards of apigenin, luteolin and, quercetin were obtained from Sigma-Aldrich (Madrid, Spain), and individual stock solutions (500–1000 mg L^−1^) were prepared in MeOH. LC-MS grade acetonitrile (Panreac; Barcelona, Spain) and ultrapure water (Milli-Q Waters purification system; Millipore; Milford, MA, USA) were also used. We prepared calibration curves in the range of 0.5–100 µg mL^−1^ in MeOH. The calibration graphs were used to quantify the corresponding compound or (semi)quantify compounds of the same chemical family (flavonoids). Quercetin was used for compound **4**, luteolin for compounds **5** and **6**, and apigenin for compound **7**. Chromatograms were recorded at 350 nm. Peak area was plotted versus analyte concentration to construct the calibration graph.

### 3.6. Statistical Analysis

Statistical analysis was performed using SPSS Statistics software v.22 (IBM SPSS Statistics for Windows, IBM Corp., Armonk, NY, USA). Data of all analyses, in triplicate, are expressed as mean ± standard deviation. A one-way analysis of variance (ANOVA) with Tukey’s HSD post hoc test (*p* < 0.05) was used to look for statistically significant differences among results.

## 4. Conclusions

In this work, we presented the chemical composition and antioxidant activity of *Cathissa reverchonii*, a threatened species, to provide valuable information not only from the chemical point of view but also in terms of data for protection and conservation plans. This is the first report of the phytochemical profile of *Cathissa*. Samples from two different populations in Spain were collected to study the influence of the location (geography and climatologic conditions). The main compounds were flavonoid-*C*-glycosides and saponins (depending on the samples). The two populations presented different phytochemical profiles, mainly in terms of saponins, which makes clear the influence of the location and environmental conditions in the phytochemical composition. In addition, differences were also observed (quantitatively) among the same population when the climatology (water accumulation in the soil in LV2) was different. Differences between the chemical composition of *Cathissa* and *Ornithogalum* were also established, confirming that *Cathissa* is a different plant genus. Finally, the flavonoid levels and the antioxidant activity values were compared, revealing a clear correlation: the higher the flavonoid concentration, the higher the antioxidant activity.

## Figures and Tables

**Table 1:** *C. reverchonii* samples collected and ecological conditions of sampling sites.

	Sample	Location	Geographical Coordinates	Altitude (m.a.s.l.)	Slope/ Aspect	Soil	Vegetation	T (°C)	RH (%)	P (MM)	SSM
**Population 1**	ALP	Puerto Encinas Borrachas (Alpandeire, Málaga)	36°40′29.90″ N 5°12′58.10″ W	970	60°/SE	Limestone rock	Rupicolous	17.90	72.38	529	0.49
	GRA	Carretera Grazalema-Benamahoma (Grazalema, Cádiz)	36°45′29.43″ N 5°22′16.68″ W	893	90°/NE	Limestone rock	Rupicolous	18.39	65.00	460	0.48
	MON	Hundidero (Montejaque, Málaga)	36°45′1.20″ N 5°14′18.10″ W	812	70°/N	Limestone rock	Rupicolous	17.24	65.69	456	0.49
	VIL	Puerto de Las Viñas (Villaluenga del Rosario, Cádiz)	36°41′25.62″ N 5°22′19.88″ W	936	45°/E	Limestone rock	Rupicolous	18.19	69.62	511	0.48
**Population 2**	LV1	Poyo Llano (Villacarrillo, Jaén)	38° 2′35.02″ N 2°56′0.93″ W	1295	5°/W	Limestone rock/Clay	Dry grassland	16.49	57.75	361	0.45
	LV2	Afluente Aguacebas Fuente del Tajo (Villacarrillo, Jaén)	38° 3′0.02″ N 2°56′35.95″ W	1100	0°	Clay	Wet grassland	16.49	57.75	361	0.45
	LV3	Carril Senda de la Berrea (Villacarrillo, Jaén)	38° 3′11.99″ N 2°56′34.30″ W	1135	0°	Clay	Dry grassland	16.49	57.75	361	0.45

Population 1: western area, provinces of Málaga and Cádiz; population 2: eastern area, province of Jaén. m.a.s.l.: meters above sea level. T: average temperature at 2 m (°C) in 2020. RH: average relative humidity at 2 m (%) in 2020. P: average precipitation (mm) in 2020. SSM: surface soil moisture, relative water content of the top few centimeters of soil, describing how wet or dry the soil is in its topmost layer, expressed in percent saturation (the percent of soil moisture with a value of 0 indicates a completely water-free soil, and a value of 1 indicates a completely saturated soil; where surface is the layer from the surface 0 cm to 5 cm below grade). Data obtained from NASA Prediction of Worldwide Energy Resources (POWER) (https://power.larc.nasa.gov/data-access-viewer/) Accessed on 2-February-2022.

**Table 2:** Characterization of the compounds found in the analyzed extracts of *C. reverchonii*.

No.	t*_R_* (min)	Observed [M − H]^−^	Molecular Formula	Error (ppm)	Fragment Ions	Assigned Identification	ALP	GRA	MON	VIL	LV1	LV2	LV3
1	1.76	341.1091	C_12_H_22_O_11_	−0.48	179.0556, 161.0470, 119.0346, 113.0239, 101.0241, 89.0246	Disaccharide *	✓	✓	✓	✓	✓	✓	✓
2	2.59	191.0200	C_6_H_8_O_7_	−1.22	173.0093, 129.0190, 111.0086, 87.0091	Citric acid *	✓	✓	✓	✓	✓	✓	✓
3	6.21	203.0826	C_11_H_12_N_2_O_2_	0.24	186.0544, 159.0926, 142.0661, 116.0503, 74.0250	Tryptophan *	✓	✓	✓	✓	✓	✓	✓
4	9.51	625.1412	C_27_H_30_O_17_	−0.12	607.1285, 565.1206, 535.1089, 505.0986, 463.0870,415.0655, 385.0545	Quercetin-di-*C*-glucoside	✓	✓	✓	✓	✓	✓	✓
5	11.46	609.1465	C_27_H_30_O_16_	−0.51	591.1426, 519.1144, 489.1085, 399.0730, 369.0049	Lucenin-2 (luteolin-6,8-di-*C*-glucoside)	✓	✓	✓	✓	✓	✓	✓
6	16.99	447.0933	C_21_H_20_O_11_	−0.09	429.0844, 387.0596, 357.0618, 327.0519	Isoorientin (luteolin-6-*C*-glucoside)	✓	✓	✓	✓	✓	✓	✓
7	20.51	431.0985	C_21_H_20_O_10_	−0.09	341.0669, 311.0558, 283.0596	Vitexin (apigenin-8-*C*-glucoside)	✓	✓	✓	✓	✓	✓	✓
8	27.87	887.4646	C_44_H_72_O_18_	0.09	741.4062, 609.3619, 447.3191	Saponin-1	✓	✓	✓		✓	✓	✓
9	28.18	887.4643	C_44_H_72_O_18_	0.4	741.4078, 609.3636, 447.3156	Saponin-2	✓	✓	✓	✓	✓	✓	✓
10	29.39	917.3518	C_37_H_54_N_14_O_10_S_2_	−0.22	838.3987, 458.1735	Unknown	✓	✓	✓	✓	✓	✓	✓
11	30.94	803.4426	C_40_H_68_O_16_	1.01	757.4370, 611.3786, 449.3267	Saponin-3 (formate adduct)		✓	✓				
12	36.17	1065.5476	C_51_H_86_O_23_	1.16	903.4958, 757.4346, 595.3835, 433.3329	Saponin-4		✓					
13	36.41	1065.546	C_51_H_86_O_23_	2.31	903.4951, 757.4366, 595.3866, 433.3297	Saponin-5		✓		✓			
14	37.23	903.496	C_45_H_76_O_18_	−0.05	757.4407, 595.3696, 427.5059	Saponin-6		✓					
15	38.76	327.2178	C_18_H_32_O_5_	−0.3	309.2053, 291.1955, 229.1442, 211.1334	Trihydroxy-octadecadienoic acid	✓	✓	✓	✓	✓	✓	✓
16	39.05	695.3652	C_36_H_56_O_13_	−0.34	651.3744, 591.3528, 489.3220	Unknown				✓			
17	40.26	329.2334	C_18_H_34_O_5_	−0.19	311.2233, 293.2109, 229.1442, 211.1337	Trihydroxy-octadecenoic acid	✓	✓	✓	✓	✓	✓	✓

* Identified by comparison with analytical standards and/or METLIN database. See Table 1 for acronyms and information on each location.

**Table 3:** Quantification of the main compounds detected in extracts of *C. reverchonii* in methanol.

Nº	Assigned Identification	mg g^−1^ DE						
		ALP	GRA	MON	VIL	LV1	LV2	LV3
*Flavonoids*								
4	Quercetin-di-C-glucoside	1.8 ± 0.1 ^c^	2.3 ± 0.1 ^b^	1.8 ± 0.1 ^c^	2.0 ± 0.1 ^bc^	3.8 ± 0.3 ^a^	1.33 ± 0.09 ^d^	2.4 ± 0.2 ^b^
5	Lucenin-2	5.1 ± 0.3 ^d^	5.8 ± 0.4 ^cd^	6.3 ± 0.4 ^bc^	6.5 ± 0.4 ^bc^	8.9 ± 0.5 ^a^	4.8 ± 0.3 ^d^	7.2 ± 0.4 ^b^
6	Isoorientin	0.71 ± 0.05 ^a^	0.41 ± 0.03 ^c^	0.60 ± 0.04 ^b^	0.55 ± 0.04 ^b^	0.18 ± 0.01 ^d^	0.11 ± 0.01 ^d^	0.10 ± 0.01 ^d^
7	Vitexin	1.03 ± 0.06 ^a^	0.47 ± 0.03 ^c^	0.68 ± 0.04 ^b^	0.62 ± 0.04 ^b^	0.18 ± 0.01 ^d^	0.12 ± 0.01 ^d^	0.10 ± 0.01 ^d^
TIPC		8.6 ± 0.3 ^c^	9.0 ± 0.4 ^bc^	9.4 ± 0.4 ^bc^	9.7 ± 0.4 ^bc^	13.1 ± 0.6 ^a^	6.4 ± 0.3 ^d^	9.8 ± 0.4 ^b^

DE: dried extract; TIPC. total individual phenolic content; values are mean ± SD of three parallel measurements (means in the same line not sharing the same letter are significantly different at *p* < 0.05 probability level), corresponding to the letter “^a^” for the highest value.

**Table 4:** Antioxidant properties of the extracts of *C. reverchonii* *.

Antioxidant Assay	ALP	GRA	MON	VIL	LV1	LV2	LV3
ABTS radical-scavenging (g TE/100 g DE)	2.0 ± 0.3 ^b^	2.4 ± 0.3 ^ab^	2.3 ± 0.1 ^ab^	2.2 ± 0.1 ^b^	2.9 ± 0.4 ^a^	1.9 ± 0.2 ^b^	1.9 ± 0.1 ^b^
DPPH radical-scavenging (g TE/100 g DE)	1.4 ± 0.2 ^bc^	1.7 ± 0.2 ^ab^	1.3 ± 0.2 ^bc^	1.2 ± 0.2 ^bc^	2.0 ± 0.3 ^a^	1.0 ± 0.1 ^c^	1.5 ± 0.2 ^abc^

* Values expressed as means ± standard deviation (SD) of three parallel measurements; TE: Trolox equivalent. Different letters. (^a, b^ and ^c^) in the same line indicate significant differences in the extracts (*p* < 0.05), corresponding the letter “^a^” for the highest value.

## Data Availability

Data are available from corresponding author if requested.

## Sample Availability

Samples of the compounds are not available from the authors.
